# Impact of virtual agent facial emotions and attention on N170 ERP amplitude: comparative study

**DOI:** 10.3389/fnbeh.2025.1523705

**Published:** 2025-02-10

**Authors:** Luisa Kirasirova, Olga Maslova, Vasiliy Pyatin

**Affiliations:** ^1^Department of Physiology, Samara State Medical University, Samara, Russia; ^2^Department of Science, Eurasian Technological University, Almaty, Kazakhstan; ^3^Neurointerfaces and Neurotechnologies Laboratory, Neurosciences Research Institute, Samara State Medical University, Samara, Russia

**Keywords:** N170, virtual agents, facial emotions, attention, 2D, VR, EEG

## Abstract

**Introduction:**

It is known from the literature that face perception of virtual agents affects the amplitude and latency of the ERP components. However, sensitivity of the N170 component to virtual agent facial emotions, and level of attention to facial emotional expressions were not investigated in the virtual reality environment by now, which was the aim of our study.

**Methods:**

EEG recording, 2D and 3D visual testing of the neutral, happy and disgusted facial emotions of virtual agents were used. The protocol consisted of three sessions in the attentional condition of participants to each facial emotion (passive, active, and active to neutral facial emotional expression). The amplitudes of the N170 ERP were also reflected in the comparative analysis between 2D and VR.

**Results:**

In the context of virtual agent facial emotional expressions, we identified the following dynamics of the N170 amplitude: attention (passive/active) showed no signaling effect; active attention to neutral virtual agent facial emotions reduced the N170 amplitude; significant interactions were observed between the factors “emotion × attention” and “environment × attention,” but no interaction was found among all three factors.

**Conclusion:**

The immersive quality of the environment in which visual and emotional events are presented has a less pronounced effect on early-stage facial processing at N170 amplitude. Thus, our findings indicate that the N170 amplitude is primarily modulated by the emotional content and attention directed to virtual agent facial emotional expressions.

## 1 Introduction

The study of face perception using electrophysiological measurements moves from the conventional laboratory conditions in the form of two-dimensional images on screens to the most realistic virtual conditions (Kirasirova et al., 2021; Sagehorn et al., 2023a,b). This makes it possible to overcome the significant methodological, resource, and time constraints of traditional research approaches to the study of face processing. All the more so, the virtual transformation of the research environment becomes relevant in the conditions of intensive emergence of virtual agents and avatars into the metaverse (Maslova et al., 2025). So far, the number of studies of face processing in the virtual environments is very limited, from which it follows that the parameters of event-related potentials (ERP) correlates of the perception of virtual agent emotional faces remain poorly understood (Kirasirova et al., 2021). Recent studies of N170 parameters performed in the paradigm of analyzing the amplitude and latency of early and late ERPs have shown that moving from less realistic (2D) to more realistic face perception conditions of virtual agents does not affect the sensitivity of ERPs (Sagehorn et al., 2023b). This may be due to the sliding window method of investigating the dynamic characteristics of different emotions in virtual reality (VR), which has informational limitations in presenting dynamic facial expressions (Sun et al., 2025). As a result, detailed knowledge of the role of each of the basic ERP components in processing facial emotions of virtual agents and the role of attention neural systems in modulating face processing is in the initial stages of research.

Nevertheless, studies of electrophysiological measurements of face perception under the normal laboratory conditions have shown a certain logic of visual signal processing in the form of informative temporal patterns beginning 100 ms after the visual stimulus is switched on and, which are labeled as the ERP components, which was first investigated in the mid-1990s (Bentin et al., 1996). The early ERP component (N170) reflects activity of the Fusiform Face Area in the inferior temporal cortex, as it is highly sensitive to the perception of facial icons (Hillyard and Anllo-Vento, 1998; Eimer, 2000, 2011; Rossion and Jacques, 2012; Schindler and Bublatzky, 2020; Brunet, 2023; Akdeniz, 2020; Achour-Benallegue et al., 2024; Tanaka and Jiang, 2024). To date, the data on the possibility of modulating the latency and amplitude of the N170 remain contradictory in the literature. Thus, attention is believed to be among the most important factors modulating neural processing of emotional stimuli and, of particular importance in the aspect of our article, the N170 response is enhanced by active attention in the laboratory setting (Eimer, 2011). However, there is evidence, that there is no modulatory effect of attention in the early stage of face processing. Another aspect of N170 modulation is associated with emotional contextual content. Higher amplitudes of N170 were recorded in response to the presentation of pictures on the monitor screen with the negative emotions compared to the positive ones (Pourtois et al., 2013). This debate continues against the backdrop that there is no clear evidence as to which the first facial impressions demonstrate sufficient visual accuracy (Zebrowitz, 2017). Meanwhile, the interplay between emotion and attention is well-known from the early literature (Taylor and Fragopanagos, 2005). This emotional effect is reflected in the early modulation of the face-specific N170 ERP component (Blau et al., 2007). Overall, the interaction between attention and emotions occurs early in the processing of visual P200 amplitude according to the studies (Carretie et al., 2001; Schindler and Bublatzky, 2020; MacKay et al., 2024). As recent studies have shown, such interaction at the early stages of processing emotional stimuli can take place under the lateralization conditions with the participation of the right hemisphere (Hartikainen, 2021).

At present, the above logic of early facial processing contributes to the methodological resources in the study of virtual agent facial processing. However, we believe that investigating the virtual agent facial processing in the completely different VR experimental environment will create new information opportunities in the presentation of dynamic facial expressions (Sun et al., 2025). At the same time, it has been found that in the realism of computer graphics for the perception of virtual agent faces, the causal link of high-quality facial skin image and eye reflection is essential (Vaitonytë et al., 2021, 2022), and the on-virtual agent face reality has an advantage over the off-VR in this aspect. The realism of the image of virtual agents has a universal significance for studies of the perception’s neurophysiological mechanisms of virtual agents faces and its facial emotional expressions, which in general can be labeled as a category of visual stimuli for brain human interaction with the virtual agents and avatars (Fraser et al., 2024; Bai et al., 2024; Achour-Benallegue et al., 2024). Analysis of the first publications in this subject area shows us that research interest has turned primarily to electrophysiological responses in the N170 latency range (Sagehorn et al., 2023a,2023b). Analysis of latency and amplitude of the N170 in the VR conditions also confirms the involvement of this component in face perception, but, as the authors emphasize, only to a certain extent (Sagehorn et al., 2023b). At the on-VR level, under the conditions of virtual agent’s facial emotional expressions, N170 showed differences only for the neutral facial emotional expressions (Sollfrank et al., 2021). The smallest negative mean values of N170 amplitude are detected when the realistic virtual agent facial emotions of different clarity are presented, but not visual stimuli of different modality (Sagehorn et al., 2023b). According to these authors, the perception of the realistic 3D faces engages the face-specific information processing mechanisms that are not perceived to the same extent in the 2D conditions (Johnsdorf et al., 2023; Sagehorn et al., 2023a). This may be due to the fact that the 3D facial emotions representations require more of the brain’s processing of contextual and intrinsic information in the realistic VR conditions. On the other hand, perception of the virtual agent facial emotional expressions depends on the involvement of attention to the presented contextual information as an important aspect of the early face emotional processing. It is important to note, however, that the nature of facial emotional stimuli makes it difficult to limit the focus of attention due to the holistic signal processing involved in face perception (Primativo and Arduino, 2023).

Characterizing the properties of virtual agents it should be emphasized that such, corresponding to the size of physical objects, increase the convenience and efficiency of interaction (Bao et al., 2024). Finally, we note that an additive effect on ERP amplitude is related not only to stimulus size but also to emotional expressions, as shown in the 2D conditions (Ziereis and Schacht, 2024). The introduction concludes by emphasizing that studies of the virtual agent facial expressions and the impact of attention condition on N170 amplitude extend hypothetical insights into the neurophysiological mechanisms of information processing. These studies are also of interest in terms of the widespread “participation” of the virtual agent facial expressions in VR which are at an early stage of progress and extremely relevant to address the metaverse challenges based on the neurosociological paradigm by leveraging advanced methodologies like hyperscanning, interbrain synchrony and interpersonal empathy (Maslova et al., 2022). This is due, in our opinion, to the largest realistic conditions of VR. All the more reason, the facial expressions of the avatars have become a natural extension of users’ personalities in the virtual world (Miao et al., 2022). It is believed that realistic experiences are stored in richer and more interconnected engrams than are obtained in the conventional laboratory conditions (Johnsdorf et al., 2023). Finally, we hypothesize that there is a great potential in VR to extend the capabilities of the sliding window method in presenting dynamic facial expressions and exploring the dynamic characteristics of different emotions in VR. Along with this, the virtual experimental conditions in study of virtual agent facial expressions will make it possible to investigate the role of mechanisms of inhibitory control of emotional processing (Bartholomew et al., 2021; Mancini et al., 2022). Consequently, when analyzing the findings in the impact paradigm of virtual agent facial emotion and attention on N170 ERP amplitude, it is important to consider the pivotal role of scope of attention in determining the influence of emotional information on cognitive processes and, in particular, inhibitory control. To date, inhibitory control of emotional processing has not been analyzed in the study of virtual agent face perception, but this process is known to be of key importance in the electrophysiological measurements of early and late ERPs (Pires et al., 2014).

### 1.1 Hypothesis

As a result of the analysis, we formulate the working hypothesis of the study taking into account the special importance of high realism of the image of virtual agent facial expressions for studies of the perception’s neurophysiological mechanisms of virtual agents faces (Fraser et al., 2024; Bai et al., 2024; Achour-Benallegue et al., 2024). In addition to this, there is an additive effect of the facial emotional expressions on the ERP amplitude (Ziereis and Schacht, 2024). Finally, the visual processing depends on the interaction between attention and emotions (Carretie et al., 2001; Schindler and Bublatzky, 2020; MacKay et al., 2024). Our hypothesis is that N170 amplitude as a specific biomarker of the early stage of facial processing is provided by virtual agent facial expressions and the interaction between facial emotional expressions of virtual agents with different attention conditions in the users. This study aims to test this working hypothesis.

## 2 Materials and methods

### 2.1 Study design and participants

#### 2.1.1 Participants

Participant recruitment began with a public announcement at Samara State Medical University, inviting healthy male individuals to participate in the study. Interested individuals registered for the study and were scheduled for preliminary psychophysiological testing, which was conducted during daytime hours to maintain consistent conditions. During the testing sessions, each participant completed a series of standardized assessments administered individually. These assessments included the Montreal Cognitive Assessment (Nasreddine et al., 2005), the Hospital Anxiety and Depression Scale (Zigmond and Snaith, 1983; Herrmann, 1997; Bjelland et al., 2002; Gold et al., 2013), the Epworth Sleepiness Scale (Johns, 1991), and the Pittsburgh Sleep Quality Index (Buysse et al., 1989; Luca et al., 2015). Additional evaluations were performed to assess handedness and screen for visual or auditory impairments. Once testing was completed, the results were analyzed, and predefined inclusion and exclusion criteria (Table 1) were applied. Out of 48 individuals who participated in the testing phase, 30 met the inclusion criteria and were selected for the experimental group. The final sample consisted of 30 healthy male volunteers aged 19–21 years (20 ± 0.5 years). The study adhered to the ethical standards outlined in the Declaration of Helsinki and was approved by the Ethical Committee of Samara State Medical University (protocol code 195, dated 10 October 2018). All participants provided informed voluntary consent prior to the study.

**TABLE 1:** Inclusion and exclusion criteria for participant eligibility.

Test	Inclusion criteria	Exclusion criteria
Handedness	Right	Left
Montreal Cognitive Assessment scale	Between 26 and 30 (inclusive)	Between 0 and 25 (inclusive)
Hospital Anxiety And Depression Scale	Between 0 and 7 (inclusive)	Between 8 and 21 (inclusive)
Epworth Sleepiness Scale	Between 0 and 8 (inclusive)	Between 9 and 24 (inclusive)
Pittsburgh Sleep Quality Index	between 0 and 5 (inclusive)	Between 6 and 21 (inclusive)
Visual or auditory impairments	Absence	Presence
Medical conditions	Healthy	Any medical conditions that, in the researcher’s opinion, would interfere with the participant’s involvement in the study

#### 2.1.2 EEG recording

Electroencephalography (EEG) was performed using a setup based on the “10–10%” EEG system. Sixty-four ActiCap electrodes from the “VR-01030 BrainAmp Standard128” recording system (manufactured by BrainProducts, Germany) were installed, ensuring electrode contact resistance remained below 8 kΩ. The HTC Vive Eye Pro headset was placed over the EEG cap and used to deliver visual stimuli within the virtual environment. This setup allows for simultaneous EEG recording and VR stimulation, providing an ideal framework for studying the brain responses to emotional facial expressions in a controlled, immersive setting. Two electrodes (VEOG - AF7 placed below the right eye, HEOG - FT10 near the outer canthus of the right eye) were dedicated to electrooculography to detect and subsequently eliminate eye movement artifacts. The reference electrode was FCz, and the ground electrode was labeled as AFz. EEG was sampled with a digitization rate of 500 Hz. Recording was conducted under two experimental conditions: non-immersive (2D) and immersive virtual reality (VR). In the 2D condition, visual stimuli were presented on a 23.6-inch LED monitor with a resolution of 1920 × 1080 pixels, a refresh rate of 90 Hz, and a viewing angle of 170°. Participants were seated at a fixed distance of 70 cm from the monitor to ensure standardized viewing conditions across all sessions. For the VR condition, stimuli were delivered using the HTC Vive Eye Pro headset, which features dual 3.5-inch screens with a combined resolution of 2880 × 1600 pixels (1440 × 1600 per eye), a refresh rate of 90 Hz, and a field of view of 110°. The researcher operated from an adjacent laboratory equipped with a computer for EEG recording and visual stimulus presentation, with participant monitoring facilitated through an infrared camera and audio communication system.

#### 2.1.3 Visual and testing stimuli

Initially, the visual stimuli consisted of pictures of facial emotions from the FACES database (Ebner et al., 2010), which were converted into 3D virtual agents’ models using a custom-developed software tool. This tool was implemented based on the Avatar Maker Pro plugin, which generates 3D avatars from single 2D images, and is available on the Unity Asset Store (Unity Technologies). The facial emotions represented on the 2D and 3D facial emotional expressions included neutral, happy, and disgusted expressions. These facial emotional models were carefully standardized in terms of size, head tilt, spatial positioning, and color texture. To further explore the perceived characteristics of the stimuli, participants were asked to evaluate the apparent size of the faces presented in different environments (2D versus VR). This subjective evaluation aimed to assess how the field of view influenced participants’ perception. Specifically, participants responded to the question: “How large” did the face appear to you in this environment? Please rate the perceived size relative to its real-world appearance on a scale from 1 (very small) to 7 (very large).

The emotional authenticity of the 3D virtual agent’s facial emotional expressions was evaluated to provide reliable reference data for researchers. Stimuli testing involved an independent validation study conducted with 105 healthy participants, comparable in gender and age to the EEG study participants. These individuals anonymously completed an online survey via Google Forms. They were briefed about encountering a variety of faces displaying different facial expressions and were instructed to provide spontaneous, personalized impressions of each facial expression. A set of nine questions was administered, with three dedicated to each of the 3D models. Questions 1–3 explored initial impressions by asking, “Upon initial inspection, what emotion does the person in the image elicit?” Respondents provided free-form answers. Questions 4–6 aimed to identify the specific emotion facial expressions conveyed with the prompt, “Which emotion does the person in the image convey?” Response options were randomized and included Neutral, Anger, Happiness, Fear, Disgust, and Sadness. Finally, Questions 7–9 focused on categorizing the exhibited facial emotional expressions by asking, “What facial emotional expression does the person in the image exhibit?” with randomized response options of Positive, Negative, and Neutral. The questionnaire was structured to begin with open-ended responses, followed by emotion-choice inquiries, and concluded with questions categorizing emotional expressions. Among participants aged 19–21, the accuracy in identifying facial emotional expressions was 96.2% for negative facial emotions, 98.1% for neutral facial emotions, and 94.3% for positive facial emotions.

### 2.2 Study protocol (procedures)

#### 2.2.1 Trial structure and stimulus presentation

**FIGURE 1:**
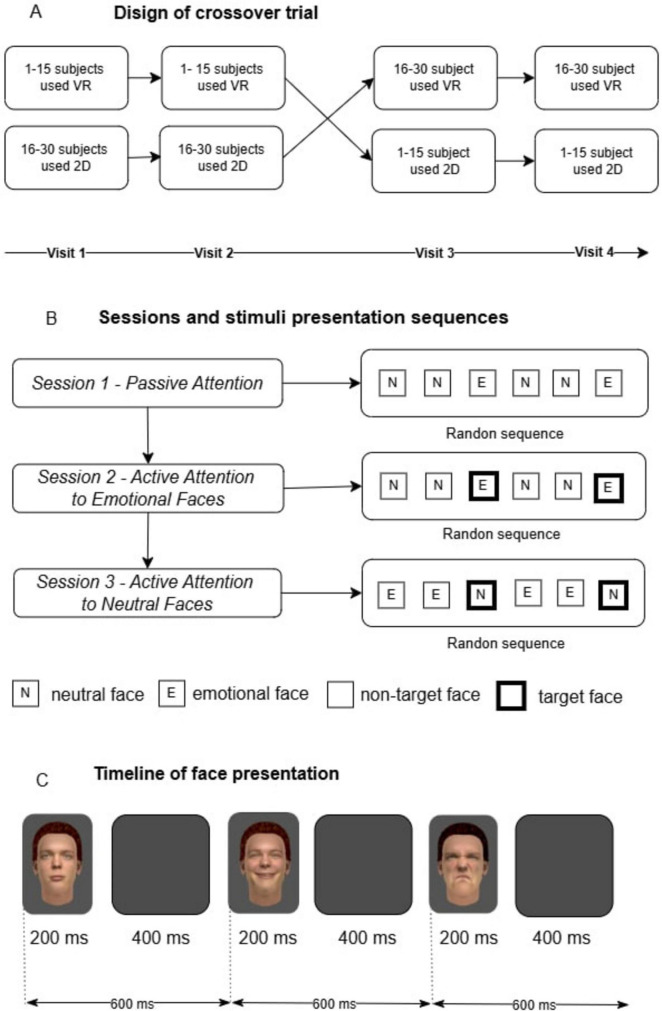
Research design outline: **(A)** Crossover study design. A total of 30 participants were divided into two groups. The first group (1–15 participants) started with the virtual reality (VR) condition, followed by the 2D condition, while the second group (16–30 participants) started with the 2D condition and then switched to VR. Each participant completed four visits, ensuring exposure to both conditions; **(B)** Sessions and stimulus presentation sequences. The experiment consisted of three sessions. In Session 1, participants passively viewed stimuli (neutral and emotional faces) presented in a randomized order. In Session 2, participants were instructed to actively attend to emotional faces (target stimuli highlighted with a black frame), while neutral faces served as non-targets. In Session 3, the focus shifted to neutral faces (target stimuli), with emotional faces becoming non-targets. Stimulus order was randomized in all sessions; **(C)** Timeline of stimulus presentation. Each face was displayed for 200 ms, followed by a blank screen for 400 ms. The total duration of one stimulus cycle was 600 ms. Stimuli appeared in a random sequence, with a fixed temporal structure maintained throughout all sessions.

The presentation of visual stimuli was conducted within the Unity environment against a dark-gray background in a randomized sequence. Each face was displayed for 200 ms, followed by a 400 ms blank screen, resulting in a total inter-stimulus interval of 600 ms. A single cycle consisted of six stimuli presented consecutively. Each recording session included 50 cycles, resulting in a total of 300 stimuli per session (Figure 1C).

#### 2.2.2 Sequence of experimental sessions

The experiment consisted of three distinct recording sessions, each targeting a different attentional condition: passive attention, active attention to facial emotional expressions, and active attention to neutral facial emotional expressions (Figure 1B).

#### 2.2.3 Session 1: passive attention

Participants were instructed to passively observe the central area of the screen or virtual scene where stimuli appeared. No specific task or response was required. Stimuli included four neutral facial emotions, one positive facial emotion, and one negative facial emotion, maintaining a 1:2 ratio of emotional to neutral stimuli.

#### 2.2.4 Session 2: active attention to facial emotions

Participants were instructed to mentally count the appearances of two target stimuli: one positive emotional expression (happiness) and one negative emotional expression (disgust). Participants were explicitly instructed to silently keep track of the number of occurrences of the two target facial expressions, without any verbal or physical response. They were asked to focus solely on these two target expressions while withholding any reactions and not counting the occurrences of neutral facial expressions. The stimuli set remained identical to Session 1, maintaining a 1:2 ratio of facial emotions to neutral facial emotional expression.

#### 2.2.5 Session 3: active attention to neutral facial emotions

Participants were instructed to mentally count the appearances of the neutral face while ignoring all emotional faces (happiness or disgust). The instruction emphasized maintaining focus exclusively on the neutral target and keeping a silent count of its occurrences throughout the session. Stimuli included four facial emotions and two neutral facial emotions, maintaining a 2:1 ratio of facial emotions to neutral facial emotional expression.

#### 2.2.6 Crossover trial design

The study employed a crossover trial design, where participants were exposed to two experimental conditions (2D and VR) in a counterbalanced sequence to minimize order effects and reduce unwanted variability arising from individual differences among participants. Participants 1–15 completed the 2D tasks first, followed by VR tasks on different days. Participants 16–30 completed the tasks in the reverse order, starting with VR and then proceeding to 2D (Figure 1A). Each participant underwent four registrations spread across different days (visits) to control for task novelty and learning effects, with sessions conducted at approximately the same time each day. This resulted in a total of 12 EEG recording sessions per participant. Of the 30 participants, 27 successfully completed all sessions; the data from three participants who withdrew from the study were excluded from the analysis.

### 2.3 Data analysis

Each EEG recording underwent pre-processing using the Brainstorm software (Tadel et al., 2011). Data transformation was conducted by changing the reference from FCz to AVERAGE Reference (Wang et al., 2023; Yao et al., 2019). EEG data underwent bandpass filtering in the range of 1.0–40.0 Hz and removal of motor and oculographic artifacts through JADE independent component analysis. The eye movement components were removed according to their topography and correlation with EOG. For the ERP analysis, the EEG fragments of 700 ms starting 100 ms before stimulus onset were used. Each epoch and channel were individually baselined by subtracting the mean of the baseline period from -50 to -2 ms before the stimulus. The N170 peak was calculated in the 150–210 ms window as the difference between facial emotions and neutral facial emotional expressions (negative-neutral, positive-neutral). The N170 amplitudes of the averaged ERPs across all sessions were separately marked for different types of stimuli. The amplitudes of N170 ERPs were homogeneous (according to the Levene’s test) and followed a normal distribution (according to the Shapiro-Wilk test). Therefore, to compare the N170 amplitudes, we utilized repeated-measures analysis of variance (RM-ANOVA) with sub-sequent pairwise comparisons corrected by the Bonferroni criterion. The statistical analysis was performed in Jamovi v 2.2 based on R-statistics.

## 3 Results

In this study, we investigated the impact of various factors, including the type of facial emotional expressions, the presentation environment, and the type of participant attention, on the amplitude of the N170 ERP. The N170 ERP, known for its occipital-parietal topography, was analyzed using the averaged amplitudes recorded at PO10 and PO9 electrodes.

### 3.1 Main factors and their interactions influencing N170 amplitude

A Repeated Measures ANOVA was conducted to investigate the impact of virtual agents’ facial emotions, attention type, and environment on the N170 potential amplitude, as well as the interactions among these factors. The analysis confirmed significant effects for the “facial emotion” factor [F(1,26) = 49.71, *p*<.001, η_*p*_^2^ = 0.657] and the “attention” factor [F(2,52) = 16.35, *p*<.001, η_*p*_^2^ = 0.386]. The impact of the “environment” factor (2D vs. VR) on N170 responses was non-significant [F(1,26) = 4.16, *p* = .052, η_*p*_^2^ = 0.138].

Regarding interactions, significant interactions were observed between the “facial emotion” and “attention” factors [F(2,52) = 4.80, *p* = .0122, η_*p*_^2^ = 0.156], as well as between the “environment” and “attention” factors [F(2,52) = 3.72, *p* = .031, η_*p*_^2^ = 0.125]. However, the interaction between the “facial emotion” and “environment” factors did not show a statistically significant impact on the change in N170 amplitude [F(1,26) = 1.30, *p* = .265, η_*p*_^2^ = 0.047], nor did the interaction of all three factors together—“facial emotion,” “environment,” and “attention” [F(2,52) = 1.47, *p* = .239, η_*p*_^2^ = 0.054].

### 3.2 Follow-up analysis of factors and interactions on N170 amplitude

To further explore the effects of the identified factors on the N170 amplitude, pairwise *post hoc* comparisons were performed with Bonferroni correction applied. These analyses aimed to delineate specific differences within and between levels of each factor, providing a more detailed understanding of the underlying patterns in the data. The results for each comparison are presented below.

#### 3.2.1 Effects of facial emotional expressions on N170 amplitude

**FIGURE 2:**
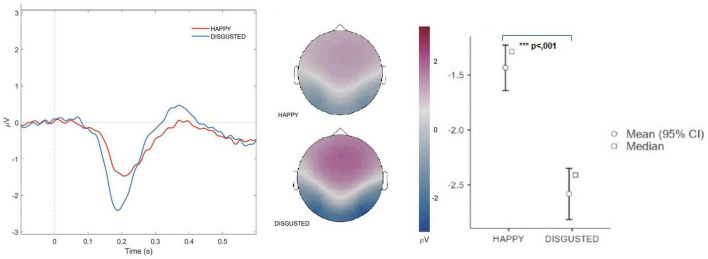
N170 amplitude for positive and negative facial emotional expressions. The graph presents the averaged N170 potentials elicited by virtual agents displaying positive (happy) facial expressions (red) and negative (disgusted) facial expressions (blue). The data reveal heightened neural sensitivity to negative facial emotions, as indicated by increased N170 amplitude for negative facial emotional expressions compared to positive ones, underscoring differential processing of facial emotional stimuli. The topographic maps at 190 ms show the scalp distribution of neural activity for both positive and negative facial emotional expressions. The maps illustrate more pronounced neural activation in occipito-temporal regions during the processing of negative facial emotional expressions, further supporting the increased sensitivity to negative facial emotional stimuli. The figure also displays mean and median plots with 95% confidence intervals, offering a clear depiction of the N170 amplitude differences between positive and negative facial emotional expressions. Level of significance: ***p < .001.

The N170 amplitude demonstrated a significant sensitivity to different facial emotional expressions. Specifically, the mean difference in amplitudes between positive (happy) and negative (disgusted) facial emotional expressions was 1.15 ± 0.163 μV. Pairwise comparisons revealed a statistically significant difference, t(26) = 7.05, *p* < 0.001. The effect size was large (Cohen’s d = 1.38), indicating a substantial distinction between the two emotional conditions. The N170 amplitude in response to negative facial emotions expressions exhibited greater negativity compared to its response to positive emotions across all experimental sessions. This suggests a robust neural differentiation in processing negative versus positive facial emotions (Figure 2).

#### 3.2.2 Effects of passive and active attention to facial emotional expressions on N170 amplitude

**FIGURE 3:**
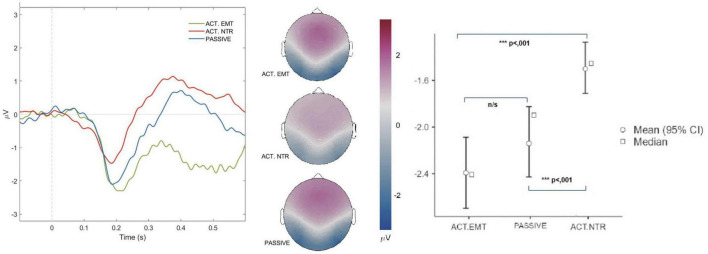
Averaged N170 potentials for virtual agents’ facial expressions across attention conditions. The graph depicts averaged N170 potentials recorded during the perception of virtual agents displaying facial emotional expressions under three distinct attention conditions: passive attention (blue), active attention to facial emotional stimuli (green), and active attention to neutral facial emotional stimuli (red). The results demonstrate a pronounced reduction in N170 amplitude during active attention to neutral virtual agent facial emotional stimuli, highlighting the modulation of neural responses according to the specific attention condition. The topographic maps at 190 ms illustrate the scalp distribution of neural activity for all three attention conditions. The maps show distinct patterns of neural activation, with reduced occipito-temporal activity during active attention to neutral virtual agent facial emotional stimuli, while activity patterns for passive attention and active attention to virtual agent facial emotional stimuli remained similar. The figure also incorporates mean and median plots with 95% confidence intervals, providing a clear representation of the data across the three attention conditions. Level of significance: ***p < .001; n/s, non-significant.

The type of attention directed by the participants also influenced the N170 amplitude. Three attention conditions were included in the study: passive attention, active attention to emotional stimuli, and active attention to neutral stimuli (Figure 3).

##### 3.2.2.1 Passive attention vs. active attention to facial emotions of virtual agents

The N170 amplitude did not show significant differences between passive attention and active attention to the facial emotional expressions. Pairwise comparisons indicated a mean difference of −0.262 ± 0.154 μV, t(26) = −1.70, *p* = .303. The effect size was small (Cohen’s d = −0.33), further suggesting that the level of attention (passive vs. active) does not substantially influence the N170 response to facial emotional expressions.

##### 3.2.2.2 Active attention to facial emotions vs. active attention to neutral facial emotions

When the participants were tasked with identifying the neutral face, the N170 amplitude for the emotional faces exhibited a significant decrease.

Comparing the N170 ERP during active attention to the facial emotional expressions and active attention to the neutral faces, a statistically significant mean difference of -0.893 ± 0.183 μV was observed, t(26) = −4.89, *p* < .001. The effect size was large (Cohen’s d = −0.94), indicating a substantial reduction in N170 amplitude during active attention to neutral faces compared to emotional faces.

##### 3.2.2.3 Passive attention vs. active attention to neutral faces

Comparing the N170 amplitude during passive attention and active attention to the virtual agent neutral facial emotion revealed a statistically significant mean difference of -0.631 ± 0.143 μV, t(26) = −4.43, *p* < .001. The effect size was large (Cohen’s d = −0.85), indicating a substantial sensitivity of the N170 amplitude to the type of attention, with a marked decrease in amplitude when the participants focused on the neutral faces compared to when they focused on the emotional faces.

#### 3.2.3 Interaction between emotion and attention on N170 amplitude in VR

**FIGURE 4:**
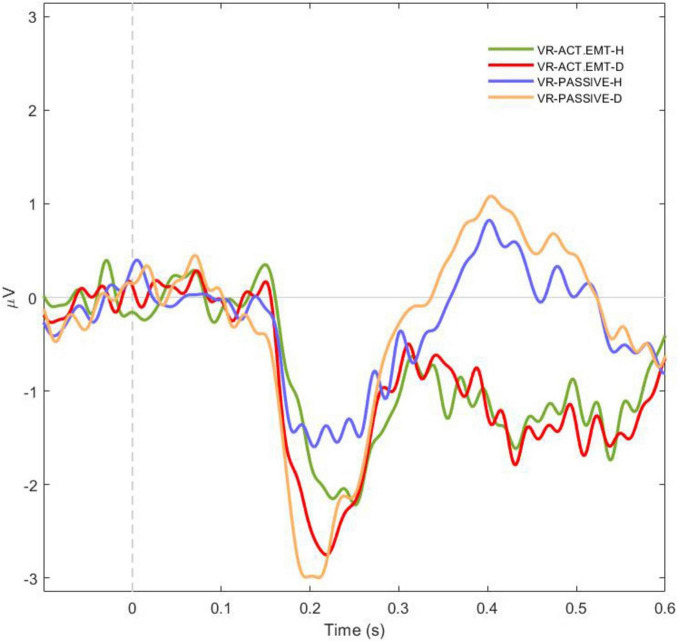
Averaged N170 potentials for facial perception across emotion and attention conditions in virtual reality (VR). The graph depicts the averaged N170 potentials recorded during the perception of facial emotional expressions with varying facial emotional expressions (H-happy or D-disgusted) under different attention conditions in a VR environment. The conditions include active attention to happy facial emotion (green), active attention to disgusted facial emotion (red), passive attention to happy facial emotion (blue), and passive attention to disgusted facial emotion (orange). The results highlight how both emotional valence and attention type modulate the N170 amplitude, with stronger responses observed for disgusted facial emotion (orange and red) compared to happy facial emotion (blue and green).

To further investigate the interaction between emotional expressions (positive vs. negative faces) and attention type (active vs. passive) on the N170 amplitude within the VR environment, a repeated measures ANOVA (RM ANOVA) was conducted, followed by *post hoc* comparisons applying the Bonferroni correction. The RM ANOVA revealed a significant main effect of the Emotion x Attention Interaction [F(3,78) = 10.2, *p* < .001, η^2^p = 0.282], indicating differences in N170 amplitude across experimental conditions (Figure 4). The *post-hoc* tests provided further insights into the interaction between emotion and attention within the immersive VR context.

The *post hoc* analysis revealed clear patterns in the interaction between emotional face and attention type on the N170 amplitude. Negative faces consistently evoked larger N170 amplitudes compared to positive faces, regardless of attention type. This pattern was observed under both active and passive attention conditions, with significant differences in amplitudes between active attention to negative faces and active attention to positive faces [t(26) = −2.85, *p* = .039, Cohen’s d = 0.55] as well as between passive attention to negative faces and passive attention to positive faces [t(26) = −4.43, *p* < .001, Cohen’s d = −0.85]. No significant differences were observed between active and passive attention conditions for positive faces [t(26) = −1.29, *p* = .577, Cohen’s d = −0.25]. Comparisons across conditions showed that the N170 amplitude was significantly larger for negative faces under active attention compared to positive faces under passive attention [t(26) = −3.47, *p* = .009, Cohen’s d = −0.67]. Additionally, no significant difference was found between active and passive attention to negative faces [t(26) = 1.45, *p* = .483, Cohen’s d = 0.28).

**FIGURE 5:**
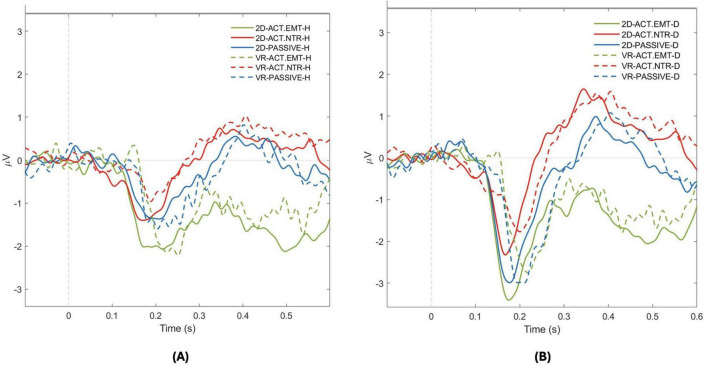
N170 Amplitude response for positive and negative facial emotional expressions across attention conditions in 2D and virtual reality (VR) environments. The graph illustrates the N170 component amplitude in response to two facial emotional expressions across different attention conditions in both 2D and VR environments: **(A)** Positive (happy- H) facial emotion; **(B)** Negative (disgusted - D) facial emotion. For each virtual agent facial emotional expressions, solid lines represent amplitudes in the 2D environment, with green line indicating active attention to facial emotional stimuli, red line for active attention to neutral facial emotion stimulus, and blue line for passive attention. The dashed lines represent the VR environment with the same color scheme: green for active emotional, red for active neutral, and blue for passive attention. This dual representation allows for a detailed comparison of neural responses to positive and negative facial emotional expressions across the environmental and attentional contexts.

The figures provided in the section “Results” visually represent the N170 ERP data under the different conditions, highlighting the differences in amplitude between various emotional stimuli, attention types, and presentation environments (Figure 5). Additional *post-hoc* analyses for all comparisons are provided in the Supplementary material. Overall, our findings suggest that the N170 ERP is robustly modulated by emotional content and attention, with less pronounced effects from the immersive quality of the presentation environment. These insights contribute to our understanding of early neural processing of emotional facial expressions and the influence of attention and environmental context on this processing.

### 3.3 Descriptive statistics

The analysis of N170 amplitudes under the varying conditions is summarized in the Table 2, where descriptive statistics provide an overview of the responses to positive and negative virtual agents’ facial emotional expressions across 2D and VR environments, as well as during the active and passive attention states. Mean, median, standard deviation, minimum, maximum, and percentile values (25th, 50th, 75th) are presented for each condition. These statistics help to illustrate the range and variability in N170 responses, highlighting the differences in amplitude associated with each attention and the facial emotion type.

**TABLE 2:** Descriptive statistics of N170 amplitudes across experimental conditions.

Parameter	Positive	Negative	2D	VR	Act. emot.	Act. neutr.	Passive
Mean	−1.43	−2.58	−2.15	−1.87	−2.39	−1.50	−2.13
Median	−1.29	−2.41	−1.97	−1.79	−2.41	−1.45	−1.89
Standard deviation	1.34	1.51	1.56	1.51	1.62	1.21	1.63
Minimum	−5.88	−6.89	−6.89	−6.20	−6.86	−4.35	−6.89
Maximum	2.53	0.649	1.60	2.53	1.60	1.18	2.53
25th percentile	−2.17	−3.37	−3.08	−2.69	−3.16	−2.27	−2.94
50th percentile	−1.29	−2.41	−1.97	−1.79	−2.41	−1.45	−1.89
75th percentile	−0.682	−1.61	−1.16	−0.814	−1.21	−0.876	−1.14

## 4 Discussion

In general, the working hypothesis of our study is confirmed by the data obtained. Transition to the more authentic VR environment represents a crucial stage of methodological progress in the study of real-life relevant facial emotions perception, as well as in the broader study of face-related ERPs using VR (Kirasirova et al., 2021; Kirasirova and Pyatin, 2022; Sagehorn et al., 2023a,b). At the same time, we believe that a comparative analysis between face processing in the 2D and VR environment has certain limitations, as these two environments are significantly different from each other. However, the studies in the laboratory settings using 2D facial emotions indicated that the N170 component is robust to changes in the attentional engagement when processing the facial emotions (Eimer et al., 2003; Schacht et al., 2012). The traditional explanation has been related to the attentional resource theory, which posits that task relevance influences the allocation of cognitive resources, and irrelevant stimuli receive less processing (Lavie, 2005; Pessoa et al., 2002).

Our study of impact of virtual agent facial emotions and user’s attention on N170 ERP amplitude used three types of emotional facial expressions (happy, disgusted and neutral) of virtual agents without changing the complexity of the emotional facial expressions, and three different attention conditions for the experimental participants. We believe that in highly realistic conditions the virtual agent facial emotional expressions contain more information related to emotion processing and extend the study of the dynamic characteristics of different emotions in VR with the involvement of specific brain neural networks (Sun et al., 2025).

Our view is supported by a study in which the decoding of emotional valence in faces can occur even for small stimuli, and additional supra-additive effects in faces may necessitate larger size ranges or dynamic stimuli that increase arousal (Ziereis and Schacht, 2024). On this basis, we suggest that the N170 amplitude dynamics established under the virtual conditions of our study could be due to additional supra-additive effects and inhibition mechanisms. Inhibition mechanisms are known to play a central role in the organization of various cognitive domains of brain activity (Pires et al., 2014).

As a result, our study in the paradigm of the relationship between virtual agent emotions, N170 amplitude and attention conditions in the VR environment realism allows a detailed analysis of the early stage of face processing. In this case, the processing of the face of the virtual agent emotions by the neural networks of the brain related to face emotional processing occurs under conditions of more information considering the participation of global face processing (Primativo and Arduino, 2023).

### 4.1 The relationship between virtual agent emotions and N170 amplitude

The rapid presentation rate (200 ms stimulus duration and 400 ms inter-stimulus interval) was specifically chosen to focus on the evaluation of early and mid-latency ERP components, such as the N170. These component ERP are typically associated with the rapid facial processing. The duration of visual stimuli was kept strictly constant, as its dynamics may differentially affect the processing of facial stimuli (Primativo and Arduino, 2023). First of all, N170 amplitude is significantly sensitive to different facial emotional expressions of virtual agents, and negative facial emotions (disgusted) eliciting greater responses than positive (happy). This result aligns with the previous research that negative facial emotions are more potent in capturing attention and eliciting stronger neural responses (Olofsson et al., 2008; Schindler and Bublatzky, 2020). Consequently, the brain can have evolved the mechanisms to prioritize the processing of negative facial emotional stimuli, leading to enhanced neural responses as observed in the N170 component (Vuilleumier, 2005). Moreover, the differential processing of positive and negative facial emotions of virtual agents at the early stages of perception underscores the importance of emotional valence in cognitive and affective processing. This robust neural differentiation supports the hypothesis that the brain allocates more resources to processing emotionally salient stimuli, particularly those with negative valence (Pourtois et al., 2013). Such findings are consistent with the negativity bias, a well-documented phenomenon where the negative events are processed more thoroughly than the positive ones (Baumeister et al., 2001).

### 4.2 N170 amplitude in 2D vs. VR environments

The N170 amplitude showed relative insensitivity to the presentation 2D vs. virtual agent facial emotions. It can be hypothesized that the brain networks involved in face emotional processing require a great deal of information related to emotion processing. The high realism of virtual agent facial emotional expressions when using the sliding window method probably needs to be supplemented by varying the complexity of emotional expressions. While VR has been shown to enhance engagement and ecological validity in various cognitive tasks (Freeman et al., 2017), our findings and a study by other authors indicate that early perceptual processing of facial emotions, as reflected by N170, remains stable across the 2D and virtual environments (Sagehorn et al., 2023b). This stability can be explained by the fundamental nature of the face processing mechanisms, which are robust and efficient regardless of the presentation environment (Rizzo et al., 2004; Bohil et al., 2011). Moreover, to some extent, this aligns with the previous studies that have shown similar neural responses to facial stimuli in different presentation formats (Diemer et al., 2015; Wieser et al., 2010).

### 4.3 The relationship between N170 amplitude, virtual agent facial emotions and attention conditions

Our findings revealed that the attention conditions (passive attention; active attention to emotional stimuli; and active attention to neutral stimuli) to the virtual agent facial expressions directed by the participants significantly influenced the N170 amplitude. The results demonstrated notable differences in the N170 amplitudes depending on the attention condition, highlighting the role of attention in the early neural responses of the virtual agent facial emotions. It is known from the literature that stimulus nature (e.g., face as stimulus) and the cognitive processes it elicits are crucial in preferential global level, at which face processing is performed more efficiently (Primativo and Arduino, 2023).

The neural networks of several brain regions (the angular gyrus, the ventral precuneus, the left posterior cingulate cortex, the right anterior superior frontal gyrus, and two face-responsive regions) are known to perform such a function, displaying distinct activation patterns for the same facial emotional expressions (Mirabella et al., 2024). In the context of analyzing the relationship between N170 amplitude, virtual agent facial emotions and attention conditions, it is important to consider the significance of mechanisms of the inhibitory control of emotional processing (Bartholomew et al., 2021; Mancini et al., 2022), which exert key importance for electrophysiological measurements of early and late ERPs (Pires et al., 2014).

#### 4.3.1 Passive attention vs. active attention to virtual agent facial emotions

We compared the N170 amplitudes in the Session 1 (passive attention) versus Session 2 (active attention) (Figure 1). The N170 amplitude showed no significant differences between passive attention and active attention to facial emotional expressions after applying the Bonferroni correction. This suggests that under VR conditions, the functional activity of neural networks for the early perceptual processing of the virtual agent facial emotions engages more attentional resources, regardless of its subjective conditions (Wang et al., 2024). Interestingly, previous research in the 2D conditions indicated that the N170 component is robust to variations in attentional engagement when processing the facial emotions (Eimer et al., 2003; Schacht et al., 2012).

#### 4.3.2 Active attention to virtual agent facial emotions vs. active attention to neutral virtual agent facial emotions

We compared the N170 amplitudes in the Session 2 (active attention) versus Session 3. However, methodologically the sessions were performed in different sequences of the virtual facial stimuli (Figure 1). When participants were tasked with identifying the neutral facial emotions, the N170 amplitude exhibited a significant decrease. We suggest that in the combination of virtual agent facial stimuli; “Neutral-Neutral-Emotion” and “Emotion-Emotion-Neutral” the reduction in the amplitude N170 to the neutral facial emotions can be explained by the involvement of a set of inhibition processes (Pires et al., 2014; Bartholomew et al., 2021), as irrelevant positive emotional information can enhance inhibitory control (Pandey and Gupta, 2022). For example, according to some authors emotional information engages significant attentional resources and, thus, limits inhibition in the neural networks (Wang et al., 2024). On the other hand, studies show that behavioral inhibition and emotion recognition in autistic children are predominantly associated with the decreased N170 amplitude (Lee and Tong, 2024).

We hypothesize that the inhibitory control for the decreasing N170 amplitude to the neutral facial emotions is evident in the Session 3 with a combination of “Emotion-Emotion-Neutral” facial stimuli. In contrast, traditionally, according to the attentional resource theory, the decrease in the N170 amplitude is explained by the fact that a task relevance influences the allocation of cognitive resources, and irrelevant stimuli receive less processing (Lavie, 2005; Pessoa et al., 2002).

#### 4.3.3 Passive attention vs. active attention to neutral virtual agent facial emotional expressions

We compared the N170 amplitudes in the Session 1 (active attention) versus Session 3. Methodologically the sessions were performed in different sequences of the virtual facial stimuli (Figure 1). This indicates that even passive viewing of facial emotions elicits a stronger N170 response compared to when the participants actively focus on the neutral facial emotional expressions. The reduction in the N170 amplitude during active attention to the neutral facial emotional expressions further underscores the impact of inhibition processes on the processing of emotional stimuli. Our results indicate that while passive and active attention to facial emotions similarly involved in early facial perceptual mechanisms. In general, these findings have important implications for understanding the neural dynamics of attentional and emotional interaction.

## 5 Conclusion

Virtual reality environment represents a crucial stage of methodological progress in the study of real-life relevant facial emotions perception, as well as in the broader study of face-related ERPs using VR (Kirasirova et al., 2021; Kirasirova and Pyatin, 2022; Sagehorn et al., 2023a,b). We have shown that within the same modality under the conditions of VR glasses, the facial emotional expressions (happiness, disgust, and neutral) manifest equally on the N170 amplitudes, regardless of the attention conditions to virtual agent facial expressions.

This study investigated the impact of attention conditions on the N170 ERP in response to the facial emotional expressions. By examining the effects of passive attention, active attention to facial emotions, and active attention to neutral facial emotional expression, we gained valuable insights into the neural mechanisms underlying the interaction between attention, emotion processing and, presumably, inhibitory control. The early visual processing of the virtual agent facial emotions remained relatively stable between passive and active modes. However, a significant reduction in the N170 amplitude was observed when participants focused on neutral facial emotional expressions, which shows, in our opinion, the role of inhibitory control at the early stage of face processing.

The results highlight, first of all, the great methodological possibilities in virtual agent facial emotional expressions and visual cognitive processing. In conclusion, this study lays the foundation for future research and applications of virtual agent facial emotional expressions in cognitive neuroscience, neurosociology and clinical practice.

## 6 Limitations and future perspective

There are several factors acknowledged as limitations in the manuscript. The first one is the exclusive participation of male participants and the narrow age range of participants which limits the generalizability of the findings across genders and restrict the applicability of the results to broader populations. Another limiting factor is exploring only two emotions happiness and disgust which prevents these results from being transferable to other potentially relevant emotions such as anger, fear, sadness, and surprise. Finally, the study limitations for generalizability of the findings are the factors such as individual differences in emotional regulation, susceptibility to affective biases, current emotional state and life experiences, which were not included in the protocol of the present study.

Future research should focus on individual differences in early emotional regulation. In addition, studying the neural correlates of attention-emotion interactions in more realistic VR conditions will provide new insights into the cognitive processes of the brain. Specifically, future research in this area will focus on exploring different types of virtual agents, varying the complexity of emotional expressions, and studying the inhibitory control. Understanding these processes may lead to more effective cognitive and clinically relevant neurotechnology, especially utilizing a new potential of VR immersion to create neurotechnology for treatments the affective disorders by manipulating attention and emotional engagement in the VR-controlled environments.

## Data Availability

The raw data supporting the conclusions of this article will be made available by the authors, without undue reservation.
