# Supplementary material for: Impact of virtual agent facial emotions and attention on N170 ERP amplitude: comparative study
Source: Front Behav Neurosci. 2025 Feb 10;19:1523705. doi: 10.3389/fnbeh.2025.1523705 (PMC11847822; doi:10.3389/fnbeh.2025.1523705)
Supplement: Supplementary file 1 [file Data_Sheet_1.pdf]

Post Hoc Comparisons

Note: HAP – happy, DIS- disgusted, P – passive attention, AE - active attention to emotional faces, AN - active attention to neutral faces, 2D – standard monitor, VR - virtual reality headset

Post Hoc Comparisons - Emotion ✻ Attention

Comparison										
**Emotion**	**Attention**		**Emotion**	**Attention**	**Mean Difference**	**SE**	**df**	**t**	**p**	$p_{bonferroni}$
HAP	AE	-	HAP	AN	-0.7997	0.233	26.0	-3.4256	0.00205	0.03072
HAP	AE	-	HAP	P	-0.5414	0.211	26.0	-2.5680	0.01633	0.24490
HAP	AE	-	DIS	AE	1.0236	0.246	26.0	4.1536	3.13e-4	0.00469
HAP	AE	-	DIS	AN	0.0368	0.226	26.0	0.1629	0.87187	1.00000
HAP	AE	-	DIS	P	1.0410	0.288	26.0	3.6127	0.00127	0.01909
HAP	AN	-	HAP	P	0.2583	0.158	26.0	1.6353	0.11403	1.00000
HAP	AN	-	DIS	AE	1.8233	0.257	26.0	7.0963	1.55e-7	2.33e-6
HAP	AN	-	DIS	AN	0.8364	0.137	26.0	6.1077	1.87e-6	2.80e-5
HAP	AN	-	DIS	P	1.8407	0.258	26.0	7.1411	1.39e-7	2.08e-6
HAP	P	-	DIS	AE	1.5650	0.209	26.0	7.4742	6.18e-8	9.27e-7
HAP	P	-	DIS	AN	0.5781	0.186	26.0	3.1009	0.00460	0.06900
HAP	P	-	DIS	P	1.5824	0.250	26.0	6.3193	1.09e-6	1.63e-5
DIS	AE	-	DIS	AN	-0.9868	0.203	26.0	-4.8523	4.96e-5	7.44e-4
DIS	AE	-	DIS	P	0.0174	0.217	26.0	0.0803	0.93665	1.00000
DIS	AN	-	DIS	P	1.0042	0.191	26.0	5.2526	1.73e-5	2.59e-4

Post Hoc Comparisons - Emotion ✻ Environment

Comparison										
**Emotion**	**Environment**		**Emotion**	**Environment**	**Mean Difference**	**SE**	**df**	**t**	**p**	$p_{bonferroni}$
HAP	2D	-	HAP	VR	-0.170	0.166	26.0	-1.02	0.3155	1.00000
HAP	2D	-	DIS	2D	1.262	0.186	26.0	6.80	3.24e-7	1.94e-6
HAP	2D	-	DIS	VR	0.863	0.212	26.0	4.07	3.85e-4	0.00231

Post Hoc Comparisons - Emotion ✻ Environment

Comparison										
**Emotion**	**Environment**		**Emotion**	**Environment**	**Mean Difference**	**SE**	**df**	**t**	**p**	$p_{bonferroni}$
HAP	VR	-	DIS	2D	1.432	0.217	26.0	6.61	5.19e-7	3.12e-6
HAP	VR	-	DIS	VR	1.033	0.197	26.0	5.25	1.73e-5	1.04e-4
DIS	2D	-	DIS	VR	-0.398	0.177	26.0	-2.25	0.0334	0.20029

Post Hoc Comparisons - Attention ✻ Environment

Comparison										
**Attention**	**Environment**		**Attention**	**Environment**	**Mean Difference**	**SE**	**df**	**t**	**p**	$p_{bonferroni}$
AE	2D	-	AE	VR	-0.4720	0.243	26.0	-1.941	0.06317	0.94753
AE	2D	-	AN	2D	-0.8611	0.237	26.0	-3.631	0.00121	0.01820
AE	2D	-	AN	VR	-1.3975	0.293	26.0	-4.766	6.24e-5	9.35e-4
AE	2D	-	P	2D	-0.5761	0.208	26.0	-2.772	0.01016	0.15238
AE	2D	-	P	VR	-0.4199	0.254	26.0	-1.652	0.11056	1.00000
AE	VR	-	AN	2D	-0.3890	0.214	26.0	-1.819	0.08039	1.00000
AE	VR	-	AN	VR	-0.9255	0.224	26.0	-4.124	3.38e-4	0.00508
AE	VR	-	P	2D	-0.1041	0.181	26.0	-0.575	0.57055	1.00000
AE	VR	-	P	VR	0.0522	0.213	26.0	0.245	0.80867	1.00000
AN	2D	-	AN	VR	-0.5365	0.214	26.0	-2.507	0.01877	0.28161
AN	2D	-	P	2D	0.2849	0.192	26.0	1.488	0.14889	1.00000
AN	2D	-	P	VR	0.4412	0.214	26.0	2.063	0.04922	0.73824
AN	VR	-	P	2D	0.8214	0.189	26.0	4.344	1.90e-4	0.00285
AN	VR	-	P	VR	0.9776	0.202	26.0	4.834	5.21e-5	7.82e-4
P	2D	-	P	VR	0.1562	0.179	26.0	0.873	0.39065	1.00000

Post Hoc Comparisons - Emotion ✻ Attention ✻ Environment

Comparison												
**Emotion**	**Attention**	**Environment**		**Emotion**	**Attention**	**Environment**	**Mean Difference**	**SE**	**df**	**t**	**p**	**$p_{bonferroni}$**
HAP	AE	2D	-	HAP	AE	VR	-0.1689	0.294	26.0	-0.575	0.57018	1.00000
HAP	AE	2D	-	HAP	AN	2D	-0.5896	0.300	26.0	-1.964	0.06025	1.00000
HAP	AE	2D	-	HAP	AN	VR	-1.1786	0.385	26.0	-3.059	0.00509	0.33620
HAP	AE	2D	-	HAP	P	2D	-0.7500	0.299	26.0	-2.510	0.01861	1.00000
HAP	AE	2D	-	HAP	P	VR	-0.5016	0.389	26.0	-1.290	0.20855	1.00000
HAP	AE	2D	-	DIS	AE	2D	1.3268	0.353	26.0	3.760	8.71e-4	0.05750
HAP	AE	2D	-	DIS	AE	VR	0.5516	0.381	26.0	1.446	0.16008	1.00000
HAP	AE	2D	-	DIS	AN	2D	0.1943	0.293	26.0	0.664	0.51262	1.00000
HAP	AE	2D	-	DIS	AN	VR	-0.2896	0.375	26.0	-0.773	0.44668	1.00000
HAP	AE	2D	-	DIS	P	2D	0.9246	0.349	26.0	2.650	0.01351	0.89166
HAP	AE	2D	-	DIS	P	VR	0.9886	0.355	26.0	2.782	0.00993	0.65506
HAP	AE	VR	-	HAP	AN	2D	-0.4207	0.201	26.0	-2.096	0.04592	1.00000
HAP	AE	VR	-	HAP	AN	VR	-1.0097	0.268	26.0	-3.771	8.47e-4	0.05591
HAP	AE	VR	-	HAP	P	2D	-0.5811	0.177	26.0	-3.288	0.00290	0.19118
HAP	AE	VR	-	HAP	P	VR	-0.3327	0.258	26.0	-1.291	0.20810	1.00000
HAP	AE	VR	-	DIS	AE	2D	1.4957	0.307	26.0	4.871	4.73e-5	0.00312
HAP	AE	VR	-	DIS	AE	VR	0.7205	0.253	26.0	2.846	0.00852	0.56216
HAP	AE	VR	-	DIS	AN	2D	0.3632	0.242	26.0	1.498	0.14622	1.00000

Post Hoc Comparisons - Emotion ✻ Attention ✻ Environment

Comparison												
**Emotion**	**Attention**	**Environment**		**Emotion**	**Attention**	**Environment**	**Mean Difference**	**SE**	**df**	**t**	**p**	$p_{bonferroni}$
HAP	AE	VR	-	DIS	AN	VR	-0.1207	0.270	26.0	-0.447	0.65831	1.00000
HAP	AE	VR	-	DIS	P	2D	1.0935	0.330	26.0	3.316	0.00270	0.17827
HAP	AE	VR	-	DIS	P	VR	1.1575	0.331	26.0	3.498	0.00171	0.11259
HAP	AN	2D	-	HAP	AN	VR	-0.5890	0.215	26.0	-2.739	0.01098	0.72477
HAP	AN	2D	-	HAP	P	2D	-0.1604	0.208	26.0	-0.772	0.44722	1.00000
HAP	AN	2D	-	HAP	P	VR	0.0880	0.221	26.0	0.399	0.69336	1.00000
HAP	AN	2D	-	DIS	AE	2D	1.9164	0.331	26.0	5.782	4.33e-6	2.85e-4
HAP	AN	2D	-	DIS	AE	VR	1.1412	0.285	26.0	4.005	4.62e-4	0.03047
HAP	AN	2D	-	DIS	AN	2D	0.7839	0.172	26.0	4.548	1.11e-4	0.00732
HAP	AN	2D	-	DIS	AN	VR	0.3000	0.251	26.0	1.193	0.24370	1.00000
HAP	AN	2D	-	DIS	P	2D	1.5142	0.297	26.0	5.104	2.55e-5	0.00168
HAP	AN	2D	-	DIS	P	VR	1.5782	0.325	26.0	4.860	4.87e-5	0.00321
HAP	AN	VR	-	HAP	P	2D	0.4286	0.235	26.0	1.827	0.07918	1.00000
HAP	AN	VR	-	HAP	P	VR	0.6771	0.271	26.0	2.502	0.01897	1.00000
HAP	AN	VR	-	DIS	AE	2D	2.5054	0.356	26.0	7.036	1.80e-7	1.19e-5
HAP	AN	VR	-	DIS	AE	VR	1.7302	0.303	26.0	5.716	5.14e-6	3.39e-4
HAP	AN	VR	-	DIS	AN	2D	1.3729	0.257	26.0	5.349	1.34e-5	8.85e-4
HAP	AN	VR	-	DIS	AN	VR	0.8890	0.173	26.0	5.126	2.41e-5	0.00159
HAP	AN	VR	-	DIS	P	2D	2.1032	0.280	26.0	7.503	5.77e-8	3.81e-6
HAP	AN	VR	-	DIS	P	VR	2.1672	0.296	26.0	7.312	9.15e-8	6.04e-6
HAP	P	2D	-	HAP	P	VR	0.2485	0.272	26.0	0.912	0.36998	1.00000

Post Hoc Comparisons - Emotion ✻ Attention ✻ Environment

Comparison												
**Emotion**	**Attention**	**Environment**		**Emotion**	**Attention**	**Environment**	**Mean Difference**	**SE**	**df**	**t**	**p**	**$p_{bonferroni}$**
HAP	P	2D	-	DIS	AE	2D	2.0768	0.277	26.0	7.497	5.85e-8	3.86e-6
HAP	P	2D	-	DIS	AE	VR	1.3016	0.192	26.0	6.763	3.54e-7	2.34e-5
HAP	P	2D	-	DIS	AN	2D	0.9443	0.240	26.0	3.942	5.44e-4	0.03588
HAP	P	2D	-	DIS	AN	VR	0.4604	0.229	26.0	2.009	0.05498	1.00000
HAP	P	2D	-	DIS	P	2D	1.6746	0.265	26.0	6.327	1.07e-6	7.04e-5
HAP	P	2D	-	DIS	P	VR	1.7386	0.273	26.0	6.379	9.34e-7	6.16e-5
HAP	P	VR	-	DIS	AE	2D	1.8283	0.378	26.0	4.840	5.12e-5	0.00338
HAP	P	VR	-	DIS	AE	VR	1.0531	0.304	26.0	3.468	0.00184	0.12145
HAP	P	VR	-	DIS	AN	2D	0.6958	0.271	26.0	2.565	0.01645	1.00000
HAP	P	VR	-	DIS	AN	VR	0.2119	0.312	26.0	0.679	0.50297	1.00000
HAP	P	VR	-	DIS	P	2D	1.4261	0.339	26.0	4.203	2.75e-4	0.01816
HAP	P	VR	-	DIS	P	VR	1.4901	0.337	26.0	4.426	1.53e-4	0.01009
DIS	AE	2D	-	DIS	AE	VR	-0.7752	0.315	26.0	-2.462	0.02077	1.00000
DIS	AE	2D	-	DIS	AN	2D	-1.1325	0.306	26.0	-3.702	0.00101	0.06672
DIS	AE	2D	-	DIS	AN	VR	-1.6164	0.288	26.0	-5.616	6.66e-6	4.39e-4
DIS	AE	2D	-	DIS	P	2D	-0.4022	0.282	26.0	-1.425	0.16596	1.00000
DIS	AE	2D	-	DIS	P	VR	-0.3382	0.276	26.0	-1.227	0.23099	1.00000
DIS	AE	VR	-	DIS	AN	2D	-0.3573	0.310	26.0	-1.151	0.26027	1.00000
DIS	AE	VR	-	DIS	AN	VR	-0.8412	0.244	26.0	-3.450	0.00193	0.12721

Post Hoc Comparisons - Emotion ✻ Attention ✻ Environment

Comparison												
**Emotion**	**Attention**	**Environment**		**Emotion**	**Attention**	**Environment**	**Mean Difference**	**SE**	**df**	**t**	**p**	**$p_{bonferroni}$**
DIS	AE	VR	-	DIS	P	2D	0.3730	0.297	26.0	1.255	0.22056	1.00000
DIS	AE	VR	-	DIS	P	VR	0.4370	0.302	26.0	1.447	0.15976	1.00000
DIS	AN	2D	-	DIS	AN	VR	-0.4839	0.260	26.0	-1.861	0.07416	1.00000
DIS	AN	2D	-	DIS	P	2D	0.7303	0.241	26.0	3.034	0.00542	0.35769
DIS	AN	2D	-	DIS	P	VR	0.7943	0.313	26.0	2.536	0.01757	1.00000
DIS	AN	VR	-	DIS	P	2D	1.2142	0.239	26.0	5.083	2.70e-5	0.00178
DIS	AN	VR	-	DIS	P	VR	1.2782	0.221	26.0	5.796	4.17e-6	2.76e-4
DIS	P	2D	-	DIS	P	VR	0.0640	0.219	26.0	0.292	0.77230	1.00000
